# Study on the Function and Mechanism of miR-585-3p Inhibiting the Progression of Ovarian Cancer Cells by Targeting FSCN1 to Block the MAPK Signaling Pathway

**DOI:** 10.1155/2022/1732365

**Published:** 2022-05-13

**Authors:** Yiming Jia, Jingru Li, Jinfeng Wang, Tianjiao Luo, Xiaoqian Jing, Hongmin Zhao

**Affiliations:** Department of Gynecology, First Affiliated Hospital of Harbin Medical University, Harbin City, Heilongjiang Province 150001, China

## Abstract

Ovarian cancer (OC) is the leading cause of death for women diagnosed with gynecological cancer. Studies have shown that dysregulated miRNA expression is related to various cancers, including OC. Here, we aimed to explore the biological function and mechanism of miR-585-3p in the occurrence and development of OC. The expression level of miR-585-3p was found to be low in OC tissues and cells. We analyzed the biological function of miR-585-3p in OC through in vitro cell experiments. The results indicated that overexpression of miR-585-3p inhibited the proliferation, invasion, and migration of SW626 cells, while low expression of miR-585-3p had the opposite effect in SKOV3 cells. We then screened the target genes of miR-585-3p through miRDB database and detected the expression of target genes in OC cells. FSCN1 was found to be most significantly upregulated in OC cells. Dual-luciferase reporter assays revealed FSCN1 as a potential target of miR-585-3p. Western blot analysis showed that miR-585-3p targeted FSCN1 to inhibit protein phosphorylation of ERK. In vivo animal experiments also confirmed that miR-585-3p targets FSCN1 to inhibit tumor growth and block the MAPK signaling pathway. In summary, miR-585-3p inhibits the proliferation, migration, and invasion of OC cells by targeting FSCN1, and its mechanism of action may be achieved by inhibiting the activation of the MAPK signaling pathway. miR-585-3p may serve as a potential biomarker and therapeutic target for OC.

## 1. Background

Ovarian cancer (OC) is one of the deadliest gynecological malignances in the world, causing more than 140,000 deaths yearly [1, 2]. The five-year survival rate of OC patients with early detection is about 90% [3]. However, about 70% of patients with OC are diagnosed as advanced, with a poor prognosis [4]. Despite advances in surgery, chemotherapy, and radiotherapy, the overall survival rate of OC has not improved in the past few decades [5, 6]. The pathogenesis of OC is still largely unknown [7]. Therefore, it is urgent to study the potential mechanism of OC and find new therapeutic targets to improve the clinical prognosis of patients with OC [8, 9].

MicroRNAs (miRNAs) are a class of small noncoding RNAs composed of approximately 21-23 nucleotides in length, which regulate gene expression at the transcription or posttranscription level [10–12]. Lu et al. were able to successfully classify poorly differentiated tumors using miRNA expression profiles, demonstrating the potential of miRNA profiling in cancer diagnosis [13]. Furthermore, miRNAs may function in either a tumor suppressor or oncogenic role, depending on the function of their target. For example, miR-552 promoted the proliferation, migration, and invasion of OC by regulating the PTEN pathway [14]. The expression of miR-4429 was downregulated in OC tissues, and its level was closely related to the metastasis and prognosis of OC patients, and by negatively regulating YOD1 levels, miR-4429 inhibited the malignant development of OC [15]. These examples highlight the key role of miRNAs in OC malignancy and cancer progression.

miRNAs play an important role in a variety of biological processes, including cell proliferation, apoptosis, differentiation, and tumor migration [16]. Previous studies have shown that miR-585-3p can promote or suppress cancer in certain cancers [17, 18]. The expression of miR-585 was low in gliomas, and the overexpression of miR-585 inhibited the proliferation of glioma cells in vitro, while its gene knockout promoted the proliferation of glioma cells [19]. The expression of miR-585 was downregulated in non-small-cell lung cancer. Overexpression of miR-585 inhibited cell proliferation, migration, and invasion by targeting the 3′-untranslated region (UTR) of HSMG-1 gene [20]. These results revealed that miR-585-3p plays an important role in OC. However, there is no unified conclusion on the role of miR-585-3p in OC. Fascin actin-binding protein 1 (FSCN1) is a member of the Fascin family that encodes actin-binding proteins [21]. Recent studies have shown that fascin plays a key role in cell migration, movement, adhesion, and cell interaction [22, 23]. Upregulation of FSCN1 protein expression has been found in many cancers, such as esophageal cancer [24], non-small-cell lung cancer [25], breast cancer [26], gastric cancer [27], pancreatic ductal adenocarcinoma [28], and adrenal cortical cancer [29], so we speculate that FSCN1 may play an important role in the malignant transformation of OC [30]. However, there are few studies on the relationship between miR-585-3p and FSCN1 and its role in OC.

This study explored the expression level and biological functions of miR-585-3p and its target genes in OC through bioinformatics and molecular biology methods. The results of the study indicate that miR-585-3p was downregulated in OC. In addition, we found that miR-585-3p was involved in the progression of OC, and miR-585-3p significantly inhibited the proliferation and metastasis of OC cells. It was worth noting that FSCN1 has been proved to be a direct target of miR-585-3p, and its level decreased when miR-585-3p was overexpressed. In addition, our results also showed that miR-585-3p affected the protein phosphorylation of ERK by targeting FSCN1. The results of this paper emphasize the importance of miR-585-3p in inhibiting the proliferation, migration, and invasion of OC cells. This study provides a theoretical basis for the treatment of OC.

## 2. Methods

### 2.1. Tissue Specimen

Samples from tumors and paired normal adjacent tissues were collected from OC patients (*n* = 50) who underwent initial surgery at the First Affiliated Hospital of Harbin Medical University. All samples were snap-frozen in liquid nitrogen immediately after excision and then kept in storage. All OC cases were clinically and pathologically confirmed and staged according to the 2009 International Union for Cancer Control TNM (7th edition) classification of malignant tumors. All patients had not received radiotherapy, chemotherapy, or other immunobiological therapy before surgery and signed a written informed consent. The utilization of these samples was approved by the Ethical Committee of the First Affiliated Hospital of Harbin Medical University.

### 2.2. Cell Culture and Transfection

The human OC cell lines (SKOV3 and SW626) and the normal human ovarian cells IOSE-80 were purchased from the American Type Culture Collection (Manassas, VA, USA). All cell lines were maintained in RPMI-1640 supplemented with 10% fetal bovine serum (all Gibco; Thermo Fisher Scientific, Inc., Waltham, MA, USA), 100 mg/ml penicillin, and 100 mg/ml in a humidified atmosphere of 5% CO_2_ at 37°C. In order to explore the function of miR-585-3p in OC, SW626 cells were transfected with miR-585-3p mimic or the negative scrambled control (NC), and SKOV3 cells were transfected with miR-585-3p inhibitor or the negative scrambled control (NC). Small interfering (si)RNA NC inhibitor was purchased from GenePharma, Inc. (Sunnyvale, CA, USA). Cell transfection was performed using Lipofectamine™ 2000 (Invitrogen; Thermo Fisher Scientific, Inc.), according to the manufacturer's protocol.

### 2.3. Quantitative Real-Time Polymerase Chain Reaction (qRT-PCR)

Total RNA was extracted with TRIzol™ reagent from the cultured cells, and the RNA concentration was measured by NanoDrop One/OneC (Thermo Fisher Scientific, USA). miRNA and mRNA were reverse transcripted with All-in-One™ miRNA First-Strand cDNA Synthesis Kit (GeneCopoeia, USA) and Maxima First-Strand cDNA Synthesis Kit (Thermo Fisher Scientific, USA), respectively. Mature miRNA and mRNA were detected by qRT-PCR using AriaMx real-time fluorescent quantitative PCR system (Agilent, USA). GAPDH or/and U6 were used as endogenous controls, and the relative expression of miRNA/mRNA was calculated by a 2^−*ΔΔ*Ct^ method. The primer sequence is shown in Table 1.

### 2.4. Cell Counting KIT-8 (CCK-8)

CCK-8 was used to evaluate the cell viability. Briefly, 5 × 10^3^ cells were planted into 96-well plate and precultured overnight. 0, 24, 48, and 72 h after transfection of plasmids, we added 10 *μ*l CCK8 into the medium and detected the absorbance at 450 nm within 1.5-4 h by full wavelength multifunctional enzyme labeling instrument.

### 2.5. Scratch Wound Healing Assays

The migration ability of OC cells was measured with scratch wound healing assays. In brief, the OC cells (1 × 10^6^) cells were seeded in 6-well plate and cultured with RPMI-1640 supplemented with 10% FBS overnight. We drew three parallel lines on the back of the 6-well plate and then used a 10 *μ*l pipette tip perpendicular to the parallel line to scratch a wound. The cells were washed with PBS to remove the floating cells and then cultured in fresh medium. The cell invasion images were captured by inverted microscope.

### 2.6. Transwell Assays

Transwell assay was carried out to assess the invasive ability of SKOV3 and SW626 cells. 2 × 10^5^ transfection cells cultured without FBS were inoculated on Matrigel-coated upper chambers. The culture medium containing 20% FBS was added into the lower chambers and incubated for 24 h, and then, the uninvaded cells were removed. After that, the filter was fixed with 4% paraformaldehyde for 20 min and dyed with crystal violet for 15 min at 25°C. Five random fields in each chamber were counted by inverted microscope.

### 2.7. Western Blot Assays

After transfection, the cells were washed with ice-cold PBS three times and lysed with radioimmunoprecipitation assay lysis buffer (Beyotime Institute of Biotechnology, Haimen, China) supplemented with protease inhibitors (Promega Corp.). Thirty micrograms of proteins was subjected to sodium dodecyl sulfate polyacrylamide gel electrophoresis and then transferred to the nitrocellulose membrane. The membrane was blocked with 5% nonfat milk and incubated with the primary antibody for 1.5 h. The protein band, specifically bound to the primary antibody, was detected using the FluorChem imaging system (Alpha-Innotec GmbH, Kasendorf, Germany). The primary antibodies were FSCN1, ERK, and p-ERK (both 1 : 1,000 dilution; both Santa Cruz Biotechnology, Inc., Dallas, TX, USA) and GAPDH (1 : 5000; #5174, Cell Signaling Technology).

### 2.8. Dual-Luciferase Reporter Assays

Dual-luciferase reporter assays were carried out using the Dual-Luciferase Reporter Assay System (Promega, Madison, WI, USA). Briefly, the cells were cotransfected with miR-585-3p mimics or miR-control and pMIR-reporter luciferase vector containing a specific sequence of wild-type or mutant FSCN1 fragment. The cells were collected and lysed for luciferase detection 48 h after transfection. The relative luciferase activity was normalized against the Renilla luciferase activity.

### 2.9. Tumor Xenograft Model

OC cells (5 × 10^5^) stably infected with NC mimic or miR-585-3p mimic were intracranially inoculated in male BALB/c nude mice. Six mice were included in each group. The mice were maintained in a pathogen-free facility throughout this experiment. All mice were housed and sustained under specific pathogen-free conditions, and all experiments were approved by the Use Committee for Animal Care and performed in accordance with institutional guidelines [31]. Tumor size was measured using a slide caliper and tumor volume was determined by the formula 0.5 × *A* × *B*^2^, where *A* represents the diameter of the base of the tumor and *B* represents the corresponding perpendicular value.

### 2.10. Prediction of MicroRNA Targets

Bioinformatics algorithm (miRDB, http://mirdb.org/miRDB/index.htm) [32] was used to predict the potential targets of differentially expressed miRNA.

### 2.11. Statistical Analyses

The data are expressed as the mean ± standard deviation. All experiments were repeated at least three times. Comparisons among values for all groups were performed by one-way analysis of variance. *T* test was applied for analysis of differences between two different groups. *P* < 0.05 was considered to indicate a statistically significant difference.

## 3. Results

### 3.1. Low Expression of miR-585-3p in OC

**Figure 1:**
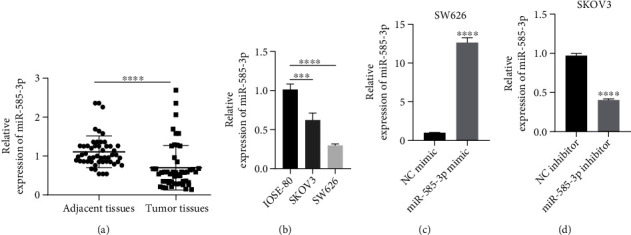
Low expression of miR-585-3p in ovarian cancer. (a) The expression of miR-585-3p in OC tissues and adjacent tissues. (b) RT-qPCR assays to detect the expression of miR-585-3p in OC cells SKOV3, SW626, and human normal ovary cell IOSE-80. (c) The relative expression of miR-585-3p in the SW626 cells after transfection of miR-585-3p overexpression. (d) The relative expression of miR-585-3p in the SKOV3 cells after transfection of miR-585-3p knockdown. ^∗∗∗^*P* < 0.001 and ^∗∗∗∗^*P* < 0.0001.

To investigate the role of miR-585-3p in OC development, we first detected the expression levels of miR-585-3p in OC tissues and adjacent tissues. As shown in Figure 1(a), the expression level of miR-585-3p in OC tissues is lower than that of adjacent tissues. Subsequently, we detected the expression of miR-585-3p in OC cell lines (SKOV3 and SW626) and human normal ovarian cell IOSE-80. The result showed that the relative expression of miR-585-3p in SKOV3 and SW626 cells was lower than that in human normal ovarian cells IOSE-80 (Figure 1(b)). The expression level of miR-585-3p was lower in SW626 cells than in SKOV3 cells. Therefore, to study the function of miR-585-3p in OC cells, we chose to perform miR-585-3p overexpression assays in SW626 cells and perform miR-585-3p knockdown assays in SKOV3 cells. The results of qRT-PCR showed that the relative expression of miR-585-3p in the miR-585-3p mimic group was higher than that in the NC mimic group in SW626 cells, and the overexpression efficiency was 12.61 times (Figure 1(c)); the relative expression level of miR-585-3p in the miR-585-3p inhibitor group was lower than that of the NC inhibitor group in SKOV3, and the interference efficiency is 0.41 times (Figure 1(d)). These results indicated that miR-585-3p was low expressed in OC tissues and cells.

### 3.2. The Overexpression of miR-585-3p Inhibits the Proliferation, Migration, and Invasion of OC Cells

**Figure 2:**
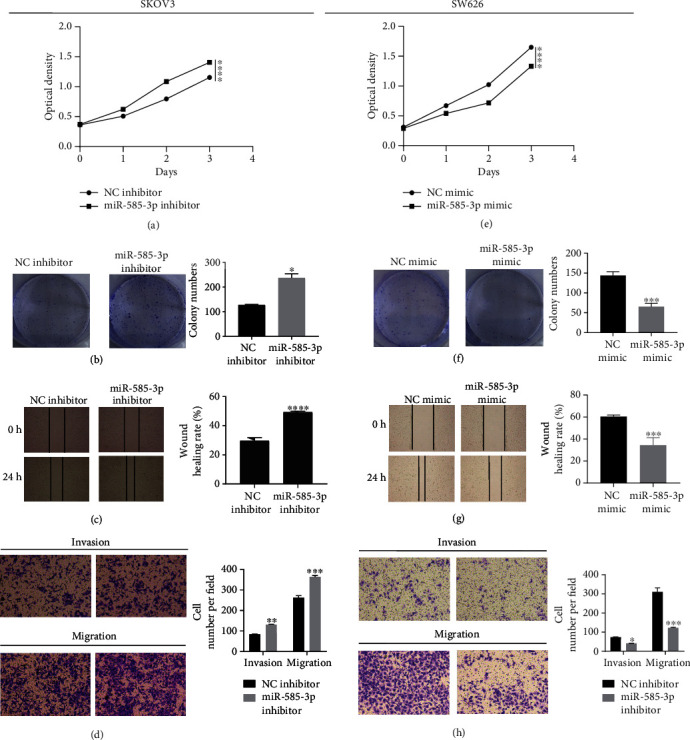
Overexpression of miR-585-3p inhibits the proliferation, migration, and invasion of OC cells. (a) CCK-8 detects the OD value of SKOV3 cells transfected with miR-585-3p inhibitor. (b) The cell clone assays detect the number of cell clones of SKOV3 cells transfected with miR-585-3p inhibitor. (c) Scratch wound healing assays detect the scratch distance after SKOV3 cells are transfected with miR-585-3p inhibitor. (d) Transwell assays detect the number of cell migration and invasion of SKOV3 cells after transfection of miR-585-3p inhibitor. (e) CCK-8 detects the OD value of SW626 cells transfected with miR-585-3p mimic. (f) The cell clone assays detect the number of cell clones of SW626 cells transfected with miR-585-3p mimic. (g) Scratch wound healing test detects the scratch distance after SW626 cells are transfected with miR-585-3p mimic. (h) Transwell assays detect the number of cell migration and invasion of SW626 cells after transfection of miR-585-3p mimic. ^∗^*P* < 0.05, ^∗∗^*P* < 0.01, ^∗∗∗^*P* < 0.001, and ^∗∗∗∗^*P* < 0.0001.

To analyze the role of miR-585-3p in OC, we conducted a series of in vitro experiments. The result of CCK-8 showed that interference with miR-585-3p expression promotes cell proliferation ability in SKOV3 cell line (Figure 2(a)). In the SW626 cell line, miR-585-3p overexpression inhibited cell proliferation (Figure 2(e)). The results of cell cloning assays showed that interference with the expression of miR-585-3p promoted cell growth in the SKOV3 cell line (Figure 2(b)), while in the SW626 cell line, overexpression of miR-585-3p inhibited the cell growth (Figure 2(f)). The results of scratch wound healing assays showed that interference with the expression of miR-585-3p promoted cell lateral migration ability in the SKOV3 cell line (Figure 2(c)), while in the SW626 cell line, overexpression of miR-585-3p inhibited the cell lateral migration (Figure 2(g)). The results of transwell assays showed that interference with miR-585-3p expression promoted cell invasion ability in SKOV3 cell line (Figure 2(d)), while in SW626 cell line, overexpression of miR-585-3p inhibited cell invasion ability (Figure 2(h)). These results confirmed that overexpression of miR-585-3p may restrain the proliferation, migration, and invasion of OC cells.

### 3.3. FSCN1 Is the Target Gene of miR-585-3p

**Figure 3:**
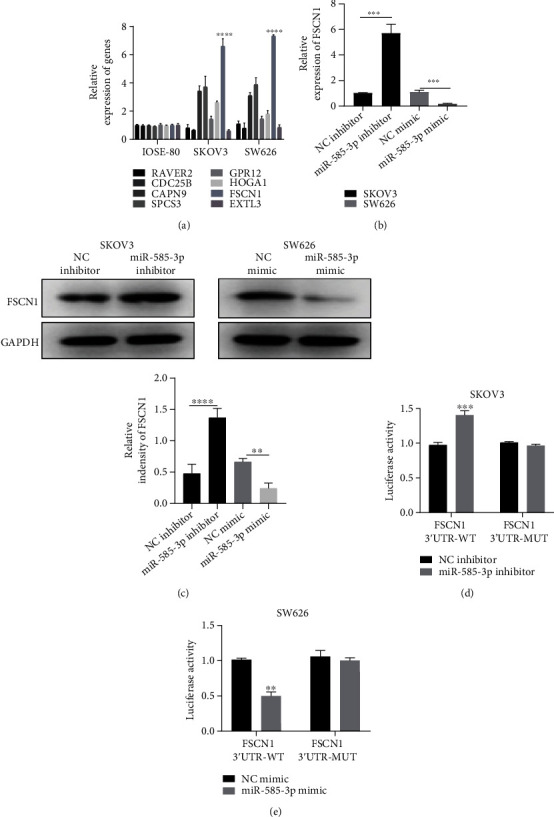
FSCN1 is the target gene of miR-585-3p. (a) qRT-PCR was used to detect the expression of target genes in OC cells and normal ovarian cells. (b) RT-PCR was used to detect the changes of FSCN1 expression after overexpression or interference of miR-585-3p. (c) Western blot was used to detect the expression of FSCN1 protein after miR-585-3p overexpression or interference. (d) The luciferase activity after cotransfection of miR-585-3p inhibitor and FSCN1 WT-3′UTR was detected by double luciferase reporter assay in SKOV3 cell line. (e) The luciferase activity after cotransfection of miR-585-3p mimic and FSCN1 WT-3′UTR was detected by double luciferase reporter assay in SW626 cell line. ^∗∗^*P* < 0.01, ^∗∗∗^*P* < 0.001, and ^∗∗∗∗^*P* < 0.0001.

Next, we attempted to identify the target genes of miR-585-3p that may be involved in OC cell expansion. Bioinformatics analysis showed that eight genes are miR-585-3p target genes, namely, YWHAQ, HSPD1, FN1, STC2, GJA1, MET, FSCN1, and FZD7. The result of qRT-PCR assays showed that the upregulation of FSCN1 was most significant in OC cells, so FSCN1 was chosen as the candidate target gene for follow-up experiments (Figure 3(a)). As shown in Figure 3(b), knockdown of miR-585-3p upregulated the mRNA expression of FSCN1 in SKOV3 cells, while in SW626 cells, overexpression of miR-585-3p downregulated the mRNA expression of FSCN1. The results of western blot assays confirmed that miR-585-3p knockdown upregulated the expression of FSCN1 protein in SKOV3 cells, while in SW626 cells, overexpression of miR-585-3p downregulated the expression of FSCN1 protein (Figure 3(c)). To further explore whether miR-585-3p directly regulates FSCN1 expression via interaction with its 3′UTR, the wild-type or mutant FSCN1 3′UTR reporter plasmids were transfected into miR-585-3p overexpression/interference OC cells and their control cells. miR-585-3p inhibitor increased luciferase activity in the FSCN1-3′UTR-WT SKOV3 cells; however, there was no significant effect in the FSCN1-3′UTR-MUT SKOV3 cells compared with that in the NC mimic group (Figure 3(d)). In addition, the miR-585-3p mimic notably decreased luciferase activity in FSCN1-3′UTR-WT SW626 cells (Figure 3(e)), while there was no effect in the FSCN1-3′UTR-MUT SW626 cells. These results suggested that FSCN1 is the target gene of miR-585-3p, and overexpression of miR-585-3p downregulates the expression of FSCN1.

### 3.4. miR-585-3p Overexpression Targets FSCN1 to Inhibit the Proliferation, Migration, and Invasion of OC Cells

**Figure 4:**
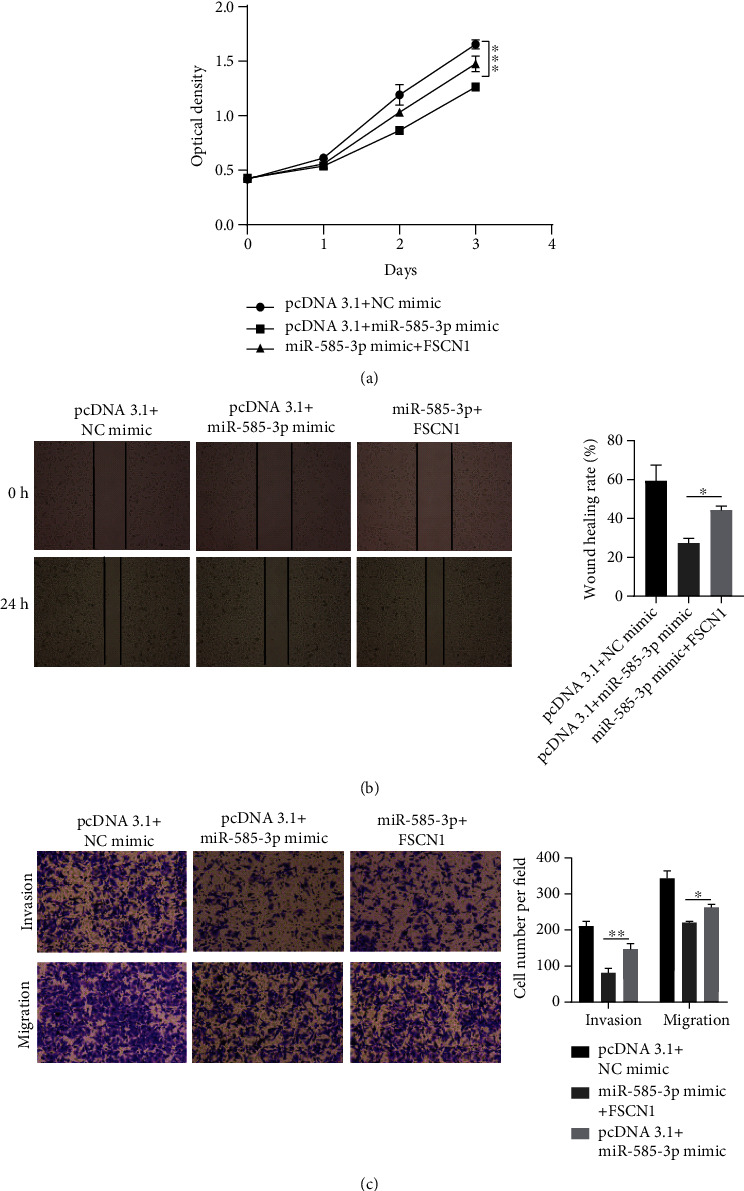
Overexpression of miR-585-3p inhibits proliferation, migration, and invasion of OC cells by targeting FSCN1. (a) RT-qPCR was used to detect the growth of OC cells after cotransfection of miR-585-3p mimic and FSCN1. (b) Scratch wound healing assays was used to detect the lateral migration ability of OC cells after cotransfection of miR-585-3p mimic and FSCN1. (c) Transwell assay was used to detect the invasive ability of OC cells after cotransfection of miR-585-3p mimic and FSCN1. ^∗^*P* < 0.05, ^∗∗^*P* < 0.01, and ^∗∗∗^*P* < 0.001.

To further verify whether miR-585-3p inhibits the proliferation, migration, and invasion of OC cells by targeting FSCN1, we carried out rescue assays. As shown in Figure 4(a), the value of OD in group miR-585-3p mimic+FSCN1 was significantly higher than that in group pcDNA3.1+miR-585-3p mimic, but lower than that in group pcDNA3.1+NC mimic, indicating that FSCN1 weakened the ability of overexpression of miR-585-3p to inhibit cell proliferation. The results of scratch wound healing assays showed that the distance between scratches in group miR-585-3p mimic+FSCN1 was larger than that in group pcDNA3.1+miR-585-3p mimic, but smaller than that in group pcDNA3.1+NC mimic, showing that FSCN1 weakened the ability of overexpression of miR-585-3p to inhibit cell horizontal migration (Figure 4(b)). The results of transwell assays showed that the number of invasive and migratory cells in group miR-585-3p mimic+FSCN1 was more than that in group pcDNA3.1+miR-585-3p mimic, but less than that that in group pcDNA3.1+NC mimic, stating that FSCN1 weakened the ability of overexpression of miR-585-3p to inhibit cell migration and invasion (Figure 4(c)). These results confirmed that FSCN1 promotes the development of OC and to a certain extent reverses the ability of miR-585-3p to inhibit the proliferation, migration, and invasion of OC.

### 3.5. Effects of miR-585-3p Targeting FSCN1 on Protein Phosphorylation of ERK

**Figure 5:**
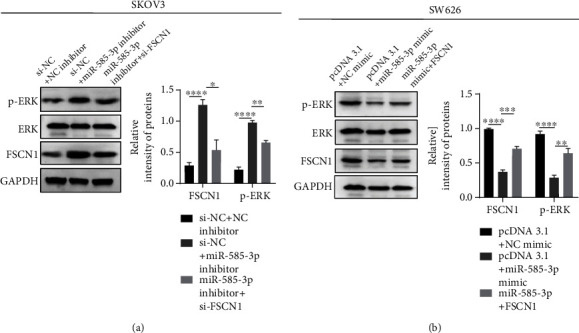
Effect of miR-585-3p targeting FSCN1 on protein phosphorylation of ERK. (a) Western blot test was used to detect the changes of p-ERK, ERK, and FSCN1 protein expression in SKOV3 cells of each group. (b) Western blot test was used to detect the changes of p-ERK, ERK, and FSCN1 protein expression in SW626 cells of each group. ^∗^*P* < 0.05, ^∗∗^*P* < 0.01, ^∗∗∗^*P* < 0.001, and ^∗∗∗∗^*P* < 0.0001.

As a member of MAPK signaling pathway, ERK signal plays an important role in regulating cell apoptosis and proliferation. To analyze the mechanism by which miR-585-3p targets FSNC1 in OC, we carried out western blot assays to analyze the expression of proteins related to ERK signaling pathway. In SKOV3 cell lines, we found that the expression of FSCN1 and p-ERK protein in si-NC+miR-585-3p inhibitor group was significantly higher than that in si-NC+NC inhibitor group, and the si-FSCN1+miR-585-3p inhibitor group was lower than that in si-NC+miR-585-3p inhibitor group (Figure 5(a)). However, in SW626 cells, the expression of FSCN1 and p-ERK protein in miR-585-3p+pcDNA3.1 group was significantly lower than that in si-NC+NC inhibitor group, and the miR-585-3p+FSCN1 group was higher than that in si-NC+miR-585-3p inhibitor group (Figure 5(b)). These results supported that miR-585-3p could block MAPK signaling pathway, which regulates the phosphorylated expression of ERK protein in OC cells, by targeting FSCN1, leading to the occurrence and development of OC.

### 3.6. Overexpression of miR-585-3p Inhibits the Proliferation of OC Tumor In Vivo

**Figure 6:**
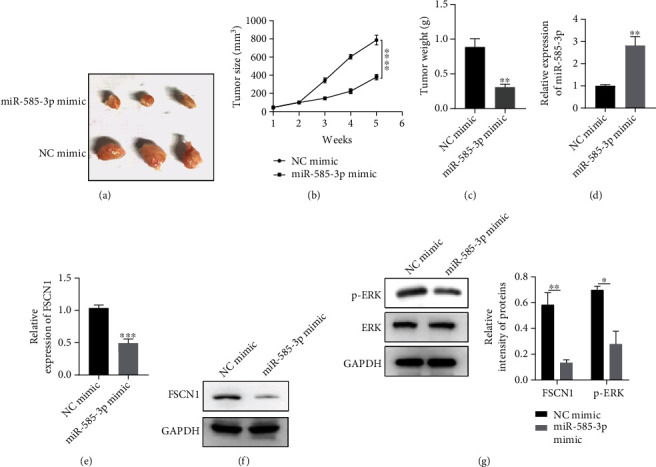
Overexpression of miR-585-3p inhibits the proliferation of OC tumor in vivo. (a) Overexpression of miR-585-3p inhibits the growth of OC tumor in vivo. (b) Overexpression of miR-585-3p restrains the growth of OC tumor volume in vivo. (c) Overexpression of miR-585-3p restrains the weight growth of OC tumor. (d) RT-qPCR was used to detect the relative expression level of miR-585-3p after OC cells were transfected with miR-585-3pmimic. (e) Overexpression of miR-585-3p inhibits FSCN1 mRNA expression. (f) Overexpression of miR-585-3p inhibits the expression of FSCN1 protein. (g) Overexpression of miR-585-3p inhibits the expression of p-ERK protein. ^∗^*P* < 0.05, ^∗∗^*P* < 0.01, ^∗∗∗^*P* < 0.001, and ^∗∗∗∗^*P* < 0.0001.

To verify the above results, we carried out the experiment of transplanting tumor in mice. As shown in Figure 6(a), the diameter of transplanted tumor in miR-585-3p mimic group was significantly smaller than that in NC mimic group. At the same time, the tumor volume and weight of miR-585-3p mimic group were significantly smaller than those of NC mimic group; with the increase of time, the transplanted tumors of the two groups gradually increased, but the growth rate of the miR-585-3p mimic group was significantly smaller than that of the NC mimic group (Figures 6(b) and 6(c)). The results of qRT-PCR assays showed that the expression of miR-585-3p in miR-585-3p mimic group was significantly higher than that in NC mimic group (Figure 6(d)); the mRNA expression of FSCN1 in miR-585-3p mimic group was significantly lower than that in NC mimic group (Figure 6(e)). The results of western blot assays showed that the expression of FSCN1 protein in miR-585-3p mimic group was significantly lower than that in NC mimic group (Figure 6(f)). And the expression of FSCN1 and p-ERK protein in miR-585-3p mimic group was significantly lower than that in NC mimic group (Figure 6(g)). These results suggested that overexpression of miR-585-3p inhibits the growth of OC, which may be achieved by downregulating the expression of FSCN1 and p-ERK.

## 4. Discussion

OC has the highest mortality among all gynecological tumors and is one of the main causes of death of gynecological malignant tumors in China [33, 34]. Increasing studies have highlighted how a multidisciplinary approach, such as a patient-centered care approach [9], the molecular genetics [35], and blood-based biomarkers [36], can improve the prognosis and survival of OC patients. Most OC patients are diagnosed clinically at an advanced stage, due to nonspecific clinical symptoms and lack of reliable early detection biomarkers [34]. Molecular analyses are of great importance for identifying diagnostic and prognostic biomarkers as well as novel therapeutic targets for OC [13, 37].

In this paper, it was found that miR-585-3p was downregulated in OC tissues and cells. A large number of studies have shown that the abnormal expression level of miRNAs is closely related to the occurrence and development of tumors [38–40]. Studies have shown that high expression of miR-96 in OC accelerates the malignant progression of OC, and its knockdown weakens the ability of proliferation and migration of OC cells by targeting FOXO3a [41]. To identify its molecular function in OC, overexpression and knockdown experiments were used to investigate the functions of miR-585-3p in the OC cell lines (SKOV3 and SW626). The results of this study revealed that upregulation of miR-585-3p could inhibit the proliferation, migration, and invasion in the SW626 cell lines. The opposite result was shown in SKOV3 cells. It is well known that miRNAs can inhibit the translation of mRNA by pairing with the 3′UTR of downstream mRNA, thus regulating the development of tumor [42–44]. In this paper, bioinformatics was used to predict that FSCN1 was a potential target of miR-585-3p, which was verified by the experimental report of double fluorescein. Rescue assays confirmed that FSCN1 could reverse the inhibitory effect of miR-585-3p on OC cells. Previous studies have confirmed that FSCN1 plays an important role in a variety of tumors, and its abnormal expression affects the development of tumors [45, 46]. FSCN1 is an actin-bundling protein that is involved in cancer metastasis and recurrence through the regulation of cellular proliferation and cloning efficiency [47]. Some scholars have shown that the overexpression of FSCN1 protein was related to the clinical malignant phenotype of non-small-cell lung adenocarcinoma, and it was a negative regulator of miR-145-5p, which inhibited the biological behaviors such as proliferation, cell cycle arrest, and apoptosis of non-small-cell lung adenocarcinoma cells [48]. It has been demonstrated that the overexpression of miR-145 could reduce bladder cancer migration through regulating FSCN1 [49]. However, it has not been reported that miR-585-3p regulates the expression of FSCN1 and affects the development of OC. The results of this study confirmed that miR-585-3p could negatively regulate the expression of FSCN1 and inhibit the proliferation, migration, and invasion of OC tumor cells. In addition, the possible molecular mechanism of miR-585-3p in OC was also studied. The results showed that miR-585-3p could target FSCN1 to inhibit ERK phosphorylation in OC cells. FSCN1 may be a novel candidate gene for the treatment of OC. ERK is a member of the MAPK signaling cascade, which includes three classes of protein kinases: MAPK kinase kinase, MAPK kinase, and MAPK [50]. Some studies have shown that miR-30a-5p improves inflammatory response through MAPK/ERK signal transduction [51]. Inhibition of FSCN1 decreases the activity of MAPK pathway, and targeting FSCN1 may inhibit the growth and metastasis of non-small-cell lung adenocarcinoma cells [52]. These studies to some extent support the relationship between FSCN1 and MAPK signaling pathway. In addition, as a new type of angiogenic factor, FSCN1 has been proved to play an important role in tumor growth and development and is a new target of antiangiogenesis therapy. Its application prospects in antitumor angiogenesis, especially in combination strategy, cannot be ignored. However, this paper does not do in-depth research on this.

## 5. Conclusion

The collective findings of the current study revealed that miR-585-3p was downregulated in OC tissues. And the overexpression of miR-585-3p targets FSCN1 to inhibit cell proliferation, migration, and invasion. The role of miR-585-3p in OC may be realized by regulating MAPK signal. miR-585-3p/FSCN1/MAPK axis may be a target for OC treatment. This study may provide new ideas and theoretical basis for clinical treatment of OC patients. Although this research provides new insights and directions, this article still has shortcomings. This study only conducted in vitro cell tests and in vivo experiments in mice, and for the verification of the MAPK signaling pathway, no activator or inhibitor was used for further verification. This is a pity for this article. In addition, whether miR-585-3p can affect the development process of OC through other signaling pathways has not been studied, which is also the direction of future research in this article.

## Figures and Tables

**Table 1:** Primer sequence details.

Gene		Sequence (5′--3′)
RAVER2	Forward primer	ACATCAGAGCTCAGTTATGGGT
	Reverse primer	GGAAGCACTGACCCTATCCC

CDC25B	Forward primer	GCGACTTGCTGCTCAAAAAGA
	Reverse primer	ACTCTTTGGGGTTTCGCTGT

CAPN9	Forward primer	CTTTTCCATCCACTGCCGGA
	Reverse primer	TCCACACAAAGGGGATCTGC

SPCS3	Forward primer	AAGCCTGTGGGTATGGGTTT
	Reverse primer	AGCCAGGCAGTTTAGCATTCA

GPR12	Forward primer	GGCTGCCTCGGGATTATTTAG
	Reverse primer	GTTCCCGAGGTACACAAGACA

HOGA1	Forward primer	GCAGGAGCTTGTCCAGGAAT
	Reverse primer	AGCTCCTCGGAAGGGGAAG

FSCN1	Forward primer	CAGCCGAACAAAGGAGCAGG
	Reverse primer	GGTCATGGTGGCAGTAGACG

EXTL3	Forward primer	AAGGCACCTGCTTGATTGCT
	Reverse primer	AATGACTTGCCCTGGAACCG

GAPDH	Forward primer	ATGTTGCAACCGGGAAGGAA
	Reverse primer	AGGAAAAGCATCACCCGGAG

U6	Forward primer	AGGGGCCGGACTCGTCATACT
	Reverse primer	GGCGGCACCACCATGTACCCT

miR-585-3p	Forward primer	UGGGCGUAUCUGUAUGCUA
	Reverse primer	GTTACAAGGTCATCCAAGAC

## Data Availability

Data supporting the discovery of this study are available from the corresponding authors on reasonable request.
